# Pregnancy Outcome Following Pelvic Infection

**DOI:** 10.1155/S1064744993000043

**Published:** 1993

**Authors:** Miklos Toth, Anu Chaudhry, William J. Ledger, Steven S. Witkin

**Affiliations:** 1 Department of Obstetrics and Gynecology Cornell University Medical College, New York NY USA; 2 Ob/Gyn Department Cornell University Medical College 525 East 68th Street, New York NY 10021 USA

## Abstract

To determine whether a previous pelvic infection has an effect on the outcome of a subsequent pregnancy, we identified women with a diagnosis of pelvic inflammatory disease (**PID**), amnionitis, and postpartum or postabortal endometritis-salpingitis by a retrospective chart review of all patients admitted to the Department of Obstetrics and Gynecology at The New York Hospital-Cornell Medical Center between 1975 and 1977 and between 1985 and 1988. Antimicrobial regimens effective against *Chlamydia trachomatis* were initiated in 1985. Controls were randomly selected patients presenting during the same time period for routine examinations who had normal Pap smears and no infections. Both groups were comparable for age, race, gravity, and parity. Differences were evaluated by chi square analysis, using the Yates correction factor. We identified 183 women with a history of the above infections who subsequently conceived, and 82 controls. There were no differences in outcome between the two index groups. Term vaginal deliveries occurred in 14.2% of the women with a prior pelvic infection and in 56% of the controls (*P* < 0.001). Among the 97 women who had had **PID**, 21 (21.6%) had a spontaneous abortion in the subsequent pregnancy, as opposed to 6 (7.3%) of the controls (*P* = 0.013). In addition, eight of the women with **PID** (but no controls) went into preterm labor (*P* = 0.021). An increased incidence of preterm labor (*P* = 0.001) was also observed in women with a history of amnionitis. A history of endometritis was not associated with an increased prevalence of abnormal outcome in subsequent pregnancies. **PID** and amnionitis may adversely affect the outcome of subsequent pregnancies.


Infectious Diseases in Obstetrics and Gynecology 1:12–15 (1993)
© 1993 Wiley-Liss, Inc.

### Pregnancy Outcome Following Pelvic Infection

**Miklos Toth, Anu Chaudhry, William J. Ledger, and Steven S. Witkin**

*Department of Obstetrics and Gynecology, Cornell University Medical College, New York, NY*

### Abstract

**To determine whether a previous pelvic infection has an effect on the outcome of a subsequent pregnancy, we identified women with a diagnosis of pelvic inflammatory disease (PID), amnionitis, and postpartum or postabortal endometritis-salpingitis by a retrospective chart review of all patients admitted to the Department of Obstetrics and Gynecology at The New York Hospital-Cornell Medical Center between 1975 and 1977 and between 1985 and 1988. Antimicrobial regimens effective against *Chlamydia trachomatis* were initiated in 1985. Controls were randomly selected patients presenting during the same time period for routine examinations who had normal Pap smears and no infections. Both groups were comparable for age, race, gravity, and parity. Differences were evaluated by chi square analysis, using the Yates correction factor. We identified 183 women with a history of the above infections who subsequently conceived, and 82 controls. There were no differences in outcome between the two index groups. Term vaginal deliveries occurred in 14.2% of the women with a prior pelvic infection and in 56% of the controls ($P < 0.001$). Among the 97 women who had had PID, 21 (21.6%) had a spontaneous abortion in the subsequent pregnancy, as opposed to 6 (7.3%) of the controls ($P = 0.013$). In addition, eight of the women with PID (but no controls) went into preterm labor ($P = 0.021$). An increased incidence of preterm labor ($P = 0.001$) was also observed in women with a history of amnionitis. A history of endometritis was not associated with an increased prevalence of abnormal outcome in subsequent pregnancies. PID and amnionitis may adversely affect the outcome of subsequent pregnancies.** © 1993 Wiley-Liss, Inc.

Key words

Pelvic inflammatory disease, amnionitis, spontaneous abortion, preterm labor

Pelvic inflammatory disease (PID) occurs in about 1 million cases annually in the United States.[1] The inability to become pregnant after PID due to occlusion of the fallopian tube is well documented.[2] However, aside from an increased prevalence of ectopic pregnancy following PID,[1,2] the effects of a previous and apparently effectively treated pelvic infection on the outcome of subsequent pregnancies have been scarcely studied.

*Chlamydia trachomatis* is a major cause of PID in developed countries.[1,2] Recent studies have also documented an association between asymptomatic chlamydial infections, as diagnosed by the presence of antichlamydial antibodies in women with no history of a symptomatic chlamydial infection, and an increased prevalence of spontaneous abortions following in vivo[3,4] and in vitro[5,6] fertilization. Reactivation of latent chlamydial infections and/or sensitization of the immune system to antigens expressed during pregnancy have been postulated to contribute to these first trimester pregnancy losses.[4,6]

To explore further the consequences of PID on subsequent pregnancies, we retrospectively examined whether women with PID, as well as with amnionitis or postabortal or postpartum endometritis, had a higher prevalence of complications during future pregnancies than did other women. In

Address correspondence/reprint requests to Dr. Miklos Toth, Ob/Gyn Department, Cornell University Medical College, 525 East 68th Street, New York, NY 10021.

Received October 20, 1992

Accepted November 24, 1992

Clinical Study

1985, recognizing the role of *C. trachomatis* in the etiology of PID, the Centers for Disease Control (CDC) first recommended inclusion of antibiotic regimens highly effective against this organism in the treatment of women with pelvic infections. To examine whether this change in management improved the prevalence of a successful outcome of the first postinfection pregnancy, we compared patients treated for pelvic infections both before and after 1985. The results suggest that women with PID had an increased prevalence of spontaneous abortion and preterm labor in the subsequent pregnancy. No differences were found, however, between groups of women with PID who were treated prior to or after implementation of the CDC guidelines for antibiotic coverage of *C. trachomatis*.

### MATERIALS AND METHODS

All women, both ward and private cases, treated in the Department of Obstetrics and Gynecology at The New York Hospital-Cornell Medical Center between 1975 and 1989 were included. Charts from two groups of women who had PID, amnionitis, or postpartum or postabortal endometritis-salpingitis were selected from a computer database of medical records and reviewed for the first obstetric event following the infection. In the first group, admitted to the hospital and treated between 1975 and 1977, 1,100 charts were reviewed and 92 cases of subsequent pregnancies were identified. In the second group, composed of patients seen between 1985 and 1988, 1,320 charts were reviewed and 91 cases were identified. The control group, consisting of 82 women, was obtained from a review of 980 charts of randomly selected patients who presented during 1975–1978 and 1985–1988 for a routine gynecological examination and who had a normal Pap smear and no infections prior to the first subsequent pregnancy. The three groups were comparable in age, race, gravity, and parity.

A term (≥37 weeks) vaginal delivery was considered a normal outcome. Spontaneous abortion was defined as loss of pregnancy before completion of the 12th gestational week. No abortions occurred in our population between the 13th and 20th weeks of gestation. Two second trimester losses occurred in both the index and control groups, at 20 and 22 weeks and 20 and 23 weeks, respectively. These were not included in the spontaneous abortion group. Preterm labor was defined as the presence of regular uterine contractions with progressive cervical dilation. Premature rupture of membranes (PROM) was defined as rupture of the membranes at least 12 hours before the onset of uterine contractions.

Differences between groups were evaluated by chi square analysis, using the Yates correction factor.

**TABLE 1. Pregnancy outcome following pelvic infections**

| Outcome | No infection | No. of women with infection (%) 1975–1977 | 1985–1988 |
|---|---|---|---|
| Term, vaginal delivery | 46 (56.1%) | 15 (16.3%) | 11 (11.5%) |
| Complications[a] | 36 (43.9%) | 77 (83.7%)* | 85 (88.5%)* |

[a]Complications include spontaneous abortion, amnionitis, PROM, preterm labor, preterm birth, and ectopic pregnancy.
*$P < 0.001$ vs. the no infection group.

### RESULTS

The outcome of the first obstetric event subsequent to an upper reproductive tract infection, and the outcome in the controls, are shown in Table 1. Among the 92 women who were treated for infections from 1975 to 1977, only 16.3% experienced a term vaginal delivery in the first postinfection pregnancy. Similarly, only 11.5% of the 96 women treated for pelvic infection between 1985 and 1988 who subsequently conceived had a term pregnancy. Differences between these two groups were not significant. In marked contrast, 56% of the 82 control pregnancies resulted in a term vaginal delivery. This outcome was significantly different from both patient groups ($P < 0.001$).

Since the two groups of patients with upper reproductive tract infections were similar in pregnancy outcome, they were combined for subsequent analyses of the relationship between infectious diagnosis and specific pregnancy complication. Results are shown in Table 2. Among the 183 patients there were 97 who had had PID, 64 with postpartum endometritis-salpingitis, 10 with postabortal endometritis-salpingitis, and 12 with amnionitis. Women with a past PID had a significantly ($P < 0.013$) increased prevalence of spontaneous abortion (21.7%) than did the controls (7.3%). Additionally, the prevalence of preterm labor was significantly greater in women with past PID (8.2%, $P = 0.021$) and past amnionitis (25%,

TABLE 2. Effect of specific pelvic infections on subsequent adverse pregnancy outcome[a]

| Pregnancy outcome | No. of women (%) | | | | |
|---|---|---|---|---|---|
| | PID (n = 97) | Postpartum endometritis (n = 64) | Postabortal endometritis (n = 10) | Amnionitis (n = 12) | None (n = 82) |
| Spontaneous abortion | 21 (21.6)* | 6 (9.4) | 2 (20.0) | 0 | 6 (7.3) |
| Preterm labor | 8 (8.2)** | 4 (6.3) | 1 (10.0) | 3 (25.0)*** | 0 |
| PROM | 7 (7.2) | 3 (4.7) | 1 (10.0) | 2 (16.7) | 2 (2.4) |
| Preterm delivery | 2 (2.1) | 0 | 0 | 0 | 0 |
| Ectopic pregnancy | 9 (9.3) | 3 (4.7) | 0 | 0 | 4 (4.9) |
| Amnionitis | 1 (1.0) | 0 | 0 | 0 | 0 |

*$P = 0.013$ vs. control.
**$P = 0.021$ vs. control.
***$P = 0.001$ vs. control.
[a]PID, pelvic inflammatory disease; PROM, premature rupture of membranes.

$P = 0.001$) than in the controls. The prevalence of pregnancy complications in women with postabortal and postpartum endometritis-salpingitis was not statistically different from the control group in this study.

In each of the three women with previous amnionitis who went into preterm labor in the subsequent pregnancy, the amnionitis was detected during term, and not preterm, labor. This excludes the possibility that a prior preterm labor, rather than the amnionitis, was the risk factor for a subsequent preterm labor.

### DISCUSSION

In the present study, women with a prior PID had a subsequent threefold higher prevalence of spontaneous abortion than did women with no history of pelvic infection. In addition, the prevalence of preterm labor was also significantly increased in women with a history of PID or amnionitis. These findings provide additional support to previous studies documenting an association between upper genital tract infection and subsequent adverse pregnancy outcome.[7] Women with serological evidence of infection with *C. trachomatis*,[3–6] a major cause of both PID and ectopic pregnancy,[1,2] had a significantly higher occurrence of spontaneous abortion than did other women. A similar association between previous ectopic pregnancy and an increased prevalence of spontaneous abortion has also been made.[8]

The mechanism relating prior pelvic infection to subsequent adverse pregnancy outcome remains to be established. In this regard, the failure to detect any improvement in pregnancy outcome following initiation of the CDC guidelines for the treatment of *C. trachomatis* suggests several mechanisms. Possible interpretations of these findings are that 1) *C. trachomatis* is not involved in the sequela; 2) the CDC guidelines were ineffective at completely eliminating *C. trachomatis* from the upper genital tract of women with PID and/or their sexual partner(s); and 3) mechanisms triggered by the initial infection but no longer requiring the continued presence of bacteria may be involved.

The first mechanism appears to be highly unlikely based on the above-mentioned associations between *C. trachomatis* and spontaneous abortions.[3–6] There is evidence in the literature consistent with the second mechanism. The ability of *C. trachomatis* to persist in the upper genital tract following standard antibiotic regimens for this organism has been known since 1980.[9] Reactivation of these subclinical or latent chlamydial infections by the hormonal and immunological changes that accompany pregnancy could lead to uterine inflammation and the subsequent expulsion of the fetus. The extensive immune system activation induced by *C. trachomatis*[10,11] could also alter immune regulatory mechanisms that normally prevent expulsion of the semi-allogeneic fetus.

Furthermore, recent studies in experimental monkeys with trachoma have demonstrated that *C. trachomatis* RNA and DNA could be identified in lesions from inflammatory sites that were negative by culture for this organism.[12] This raises the possibility that the persistence of *C. trachomatis* genetic material inside infected cells could lead to the synthesis and release of chlamydial antigens, even under conditions in which viable organisms cannot be produced. Morrison et al. have demonstrated that the addition of penicillin to in vitro cultures of *C. trachomatis* prevented the formation of elementary bodies, but a 57 kD chlamydial protein continued to be synthesized and released from infected cells.[13] This 57 kD protein has been implicated in the induction of potent inflammatory responses in animals previously sensitized to *Chlamydia*.[14]

Evidence in support of the third proposed mechanism is scant. We have previously suggested that the continued induction of interferon-γ by cells chronically infected with *C. trachomatis* could induce the expression of major histocompatibility (MHC) class 2 antigens on the surface of fallopian tube epithelial cells. This would allow these cells to present epithelial antigens to lymphocytes, resulting in immune system sensitization to epithelial antigens.[15] Recent evidence identifying the *C. trachomatis* 57 kD protein as a member of the 60 kD heat shock protein family,[13] with extensive amino acid sequence homology to the human 60 kD heat shock protein, suggests an additional mechanism. Sensitization of lymphocytes to conserved regions of the chlamydial 57 kD protein could eventually induce an autoimmune response to the human 60 kD heat shock protein.[16]

We are aware that it is difficult to reach firm conclusions based on retrospective analyses. In the present investigation, however, we considered this approach to be acceptable because of our stable patient population and the ability to perform long-term follow-ups. In any event, the present data, in conjunction with earlier studies cited above, strongly suggest that the adverse effects of pelvic infection are not limited to tubal-based infertility and an increased risk of ectopic pregnancy but also influence the outcome of subsequent intrauterine pregnancies. These studies also emphasize the need for further investigations on the possible persistence of *C. trachomatis* in the genital tract following antibiotic treatment as well as for the initiation of prospective studies to assess the effect of antecedant upper genital tract infection by *C. trachomatis* and by other microorganisms on pregnancy outcome.

### REFERENCES

1. Expert committee on pelvic inflammatory disease: Pelvic inflammatory disease. Research directions for the 1990s. Sex Transm Dis 18:46–64, 1991.
2. Westrom L, Mardh PA: Acute pelvic inflammatory disease (PID). In Holmes KK, Mardh PA, Sparling PF (eds): Sexually Transmitted Diseases. 2nd ed. New York: McGraw-Hill, pp 593–613, 1990.
3. Quinn P, Petric M, Barkin M: The prevalence of antibody to *Chlamydia trachomatis* in spontaneous abortion and infertility. Am J Obstet Gynecol 156:291–296, 1987.
4. Witkin SS, Ledger WJ: Antibodies to *Chlamydia trachomatis* in sera of women with recurrent spontaneous abortions. Am J Obstet Gynecol 167:135–139, 1992.
5. Lunenfeld E, Shapiro BS, Sarov B, Sarov I, Insler V, Decherney A: The association between *Chlamydia*-specific IgG and IgM antibodies and pregnancy outcome in an in vitro fertilization program. J In Vitro Fertil Embryo Transfer 6:222–227, 1989.
6. Licciardi F, Grifo JA, Rosenwaks Z, Witkin SS: Relation between antibodies to *Chlamydia trachomatis* and spontaneous abortion following in vitro fertilization. J Assisted Reprod Genet 9:207–210, 1992.
7. Toth M, Witkin SS, Ledger WJ, Thaler H: The role of infection in the etiology of preterm birth. Obstet Gynecol 71:723–726, 1988.
8. Fedele L, Acaia B, Parazzini F, Ricciardiello O, Candiani GB: Ectopic pregnancy and recurrent spontaneous abortion: Two associated reproductive failures. Obstet Gynecol 73:206–208, 1989.
9. Henry-Suchet J, Catalan F, Loffredo V, Serfaty D, Siboulet A, Perol Y, Sanson MJ, Debache C, Pigeau F, Coppin R, DeBrux J, Poynard T: Microbiology of specimens obtained by laparoscopy from controls and from patients with pelvic inflammatory disease or infertility with tubal obstruction: *Chlamydia trachomatis* and *Ureaplasma urealyticum*. Am J Obstet Gynecol 138:1022–1025, 1980.
10 Witkin SS, Toth M, Jeremias J, Ledger WJ: Increased inducibility of inflammatory mediators from peripheral blood mononuclear cells of women with salpingitis. Am J Obstet Gynecol 165:719–723, 1991.
11. Toth M, Jeremias J, Ledger WJ, Witkin SS: In vivo tumor necrosis factor production in women with salpingitis. Surg Gynecol Obstet 174:359–362, 1992.
12. Holland SM, Hudson AP, Bobo L, Whittum-Hudson JA, Viscidi RP, Quinn TC, Taylor HR: Demonstration of chlamydial RNA and DNA during a culture negative state. Infect Immun 60:2040–2047, 1992.
13. Morrison RP, Belland RJ, Lyng K, Caldwell HD: Chlamydial disease pathogenesis. The 57 kD chlamydial hypersensitivity antigen is a stress response protein. J Exp Med 170:1271–1283, 1989.
14. Morrison RP, Lyng K, Caldwell HD: Chlamydial disease pathogenesis. Ocular hypersensitivity elicited by a genus-specific 57-kD protein. J Exp Med 169:663–675, 1989.
15. Grifo JA, Jeremias J, Ledger WJ, Witkin SS: Interferon-γ in the diagnosis and pathogenesis of pelvic inflammatory disease. Am J Obstet Gynecol 160:26–31, 1989.
16. Morrison RP, Manning DS, Caldwell HD: Immunology of *Chlamydia trachomatis* infections. In Quinn TC (ed): Sexually Transmitted Diseases. New York: Raven Press, pp 57–84, 1992.
